# Roles for *wbtC*, *wbtI*, and *kdtA* Genes in Lipopolysaccharide Biosynthesis, Protein Glycosylation, Virulence, and Immunogenicity in *Francisella tularensis* Strain SCHU S4 

**DOI:** 10.3390/pathogens1010012

**Published:** 2012-09-10

**Authors:** Susan. M. Twine, Evguenii Vinogradov, Helena Lindgren, Anders Sjostedt, J. Wayne Conlan

**Affiliations:** 1 National Research Council Canada, Institute for Biological Sciences, 100 Sussex Drive, Ottawa, ON K1A 1L1, Canada; E-Mails: evguennii.vinogradov@nrc-cnrc.gc.ca (E.V.); wayne.conlan@nrc-cnrc.gc.ca (J.W.C.); 2 Department of Clinical Microbiology, Clinical Bacteriology, Umeå University, Umeå SE-90185, Sweden; E-Mails: helena.lindgren@climi.umu.se (H.L.); anders.sjostedt@climi.umu.se (A.S.)

**Keywords:** *Francisella tularensis*, lipopolysaccharide, glycosylation, virulence

## Abstract

Using a strategy of gene deletion mutagenesis, we have examined the roles of genes putatively involved in lipopolysaccharide biosynthesis in the virulent facultative intracellular bacterial pathogen, *Francisella tularensis* subspecies *tularensis*, strain SCHU S4 in LPS biosynthesis, protein glycosylation, virulence and immunogenicity. One mutant, *∆wbtI*, did not elaborate a long chain *O*-polysaccharide (OPS), was completely avirulent for mice, and failed to induce a protective immune response against challenge with wild type bacteria. Another mutant, *∆wbtC*, produced a long chain OPS with altered chemical and electrophoretic characteristics. This mutant showed markedly reduced glycosylation of several known glycoproteins. Additionally this mutant was highly attenuated, and elicited a protective immune response against systemic, but not respiratory challenge with wild type SCHU S4. A third mutant, *∆kdtA*, produced an unconjugated long chain OPS, lacking a detectable core structure, and which was not obviously expressed at the surface. It was avirulent and elicited partial protection against systemic challenge only.

## 1. Introduction

The gram negative coccobacillus, *Francisella tularensis*, is a facultative intracellular bacterial pathogen of multiple mammalian species including humans [1]. Two subspecies, subsp. *holarctica*, and the more virulent subsp. *tularensis*, can cause severe infection, tularemia, in humans. In particular, disseminated infection, typhoidal tularemia, following inhalation of subsp. *tularensis*, had a mortality rate of 30%–60% in the pre-antibiotic era [1]. Consequently, subsp. *tularensis* was developed as a biological warfare agent [2]. During the past several years, multiple virulence factors of *F. tularensis* have been revealed [3,4]. Loss of virulence genes results in a spectrum of attenuations and abilities of the mutant strains to elicit a protective immune response against challenge with wild type strains. Our interest has been to develop attenuated strains of subsp. *tularensis* strain, SCHU S4, which can be used as live vaccines. To this end, we have produced approximately 100 deletion mutants over the past several years including mutants missing genes involved in lipopolysaccharide (LPS) biosynthesis. The latter are the subject of the present study. 

*F. tularensis* elaborates LPS with a polymeric *O*-polysaccharide (OPS). The *O*-antigen repeat unit (–4-)-α-D-GalpNAcAN-(-1-4)-α-D-GalpNAcAN-(1-3)-ß-D-QuipNAc-(1-2)-ß-D-Quip4NFo-(1- )n, is unique to subsp. *holarctica* and subsp. *tularensis*, and is distinct from the *O*-antigen of the related, but clinically irrelevant *novicida* subspecies [5,6]. Recently, it was shown that polymerization of the O-antigen occurs via a Wzy-dependent mechanism [7]. OPS is a critical virulence factor for the pathogen, possibly by conferring resistance to killing by serum or macrophages, or by allowing stealthy infection [8]. A putative *F. tularensis* OPS biosynthesis gene locus has been proposed consisting of genes *wbtA-*to-*wbtN* encoding protein homologs of sugar epimerases, transferases, dehydrogenases, and flippases known to be involved in LPS biosythesis in other gram negative bacteria [9]. Mutants with defective *wbtA*, *wbtI*, *wbtM* genes and so called “grey variant” mutants of the empirically attenuated *holarctica* strain, LVS, lack polymeric OPS and are further reduced in virulence for mice [10,11,12,13,14]. In contrast, a *wbtC* transposon mutant of LVS expressed high molecular weight LPS with altered electrophoretic characteristics compared to wild type [13], but its relative virulence was not investigated. In subsp. *tularensis*, a *ΔwbtDEF* mutant failed to elaborate OPS and was highly attenuated [8]. In addition to OPS, LPS biosynthesis also requires conjugation of 3-deoxy-D-manno-octulosonic acid (KDO) to Lipid A to allow for subsequent attachment of the core region [15]. Mannose is the major sugar present in the *F. tularensis* LPS core [16]. 

The aim of the present study was to characterize the broad impact of three selected mutants in LPS biosynthetic genes upon bacterial virulence, immunogenicity as well as biochemical characteristics such as protein glycosylation and LPS biosynthesis. This study was carried out entirely in the highly virulent subsp *tularensis* strain SCHU S4. The results show that genes from the LPS biosynthetic pathway, in addition to contributing to LPS biosynthesis have impact upon bacterial protein glycosylation, virulence and their potential for use as live vaccines. 

## 2. Results

Deletion mutants in the current study included *ΔwbtI*, *ΔwbtC* and *ΔkdtA.* The gene product, WbtI, is annotated as a sugar transamine/perosamine synthetase, WbtC as a UDP-sugar epimerase and KdtA as a KDO transferase. Immunological, biochemical and animal virulence studies were carried out to determine the broad impact of these gene deletions. 

### 2.1. LPS Analysis

**Figure 1:**
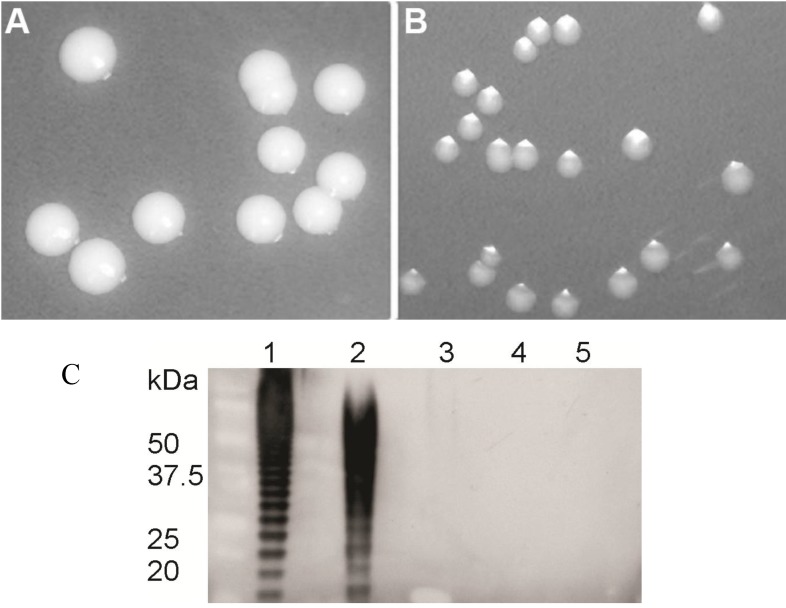
Colony morphology of wild type, (a) and *∆kdtA* (b) *F. tularensis* SCHU S4 on CHAH medium. (c) Western blots of purified lipopolysaccharide (LPS) from SCHU S4 (lane 1), *∆wbtC* (lane 2) *∆wbtI* (lane 3) *∆kdtA* (lane 4), and *F. novicida* (lane 5).

The mutants *∆wbtI* and *∆wbtC* had the same colony morphology as wild type bacteria on CHAH medium, whereas *∆kdtA* colonies were flat and dry, and lacked the characteristic green halo that encompasses normal colonies on CHAH (Figure 1a and 1b). Additionally, the *∆kdtA* colonies were much smaller, in keeping with our previous findings that this mutant grows more slowly in liquid culture [3]. Furthermore, it was recently shown that *∆kdtA* was much more sensitive than wild type bacteria to killing by normal serum (Sjostedt, manuscript submitted). Moreover, specific rabbit antiserum raised against whole bacteria only agglutinated SCHU S4 and *∆wbtC* (data not shown). Western blots were performed on purified LPS of SCHU S4, mutant strains, and *F. novicida* (Figure 1c). Only wild type and *∆wbtC* LPS were reactive, with subtly different staining patterns. Note that, in our hands when purified using hot phenol method, unconjugated *O*-antigen is not observed.

**Figure 2:**
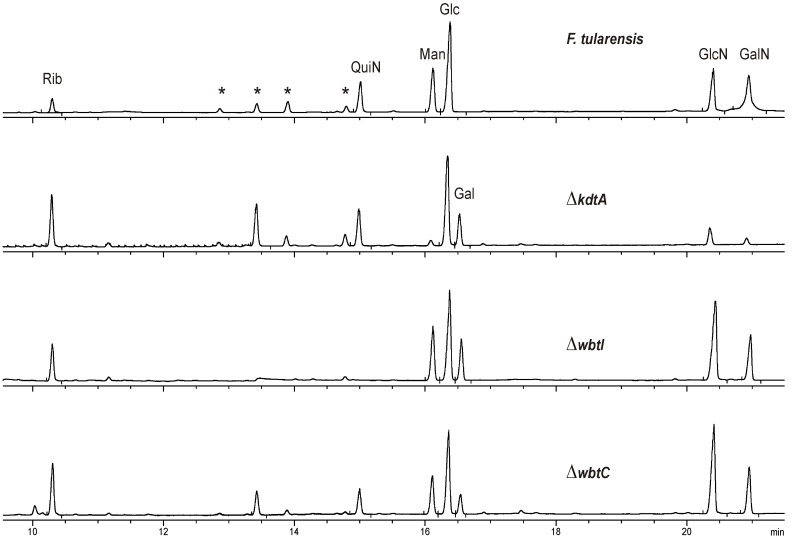
Gas chromatography traces of alditol acetates, obtained after hydrolysis of LPS. *∆wbtI* contained no QuiN, *∆kdtA* contained a very small amount of mannose, but normal amounts of the QuiN. The *∆wbtC* spectrum was similar to normal *F. tularensis.* Ribose probably originated from RNA contamination, glucose from contaminating glucans is always present in *F. tularensis* LPSpreparations. All mutants contained galactose of unknown origin. Asterisks denote components with sugar-like mass spectra, but could not be identified.

**Figure 3:**
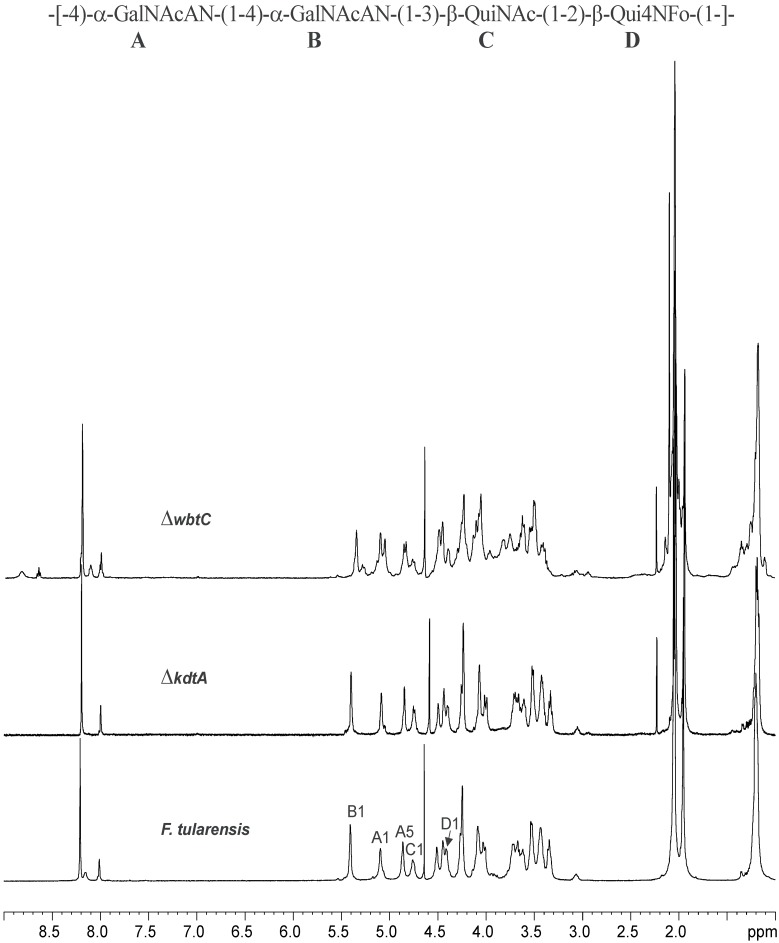
^1^H NMR spectra of *O*-antigens from *∆wbtC*, *∆kdtA* and wild-type SCHU S4 (40–45 °C, 500 MHz).

Next, chemical structural analyses were performed on the purified LPS. Gas chromatography (GC) analysis of sugar alditol acetates showed that LPS from wild type SCHU S4 and *∆wbtC* contained all of the expected sugars. However, Qui4NFm (4,6-dideoxy-4-formamido-D-glucose), a major component of normal OPS, was absent from *∆wbtI*, and mannose, a major component of the core, was essentially absent from *∆kdtA*, (Figure 2). This was confirmed by sugar analysis of whole bacteria (Figure S1). ^1^H NMR analysis of OPS (Figure 3) showed wild type and *∆kdtA* contained identical OPS structures, but *∆wbtC* spectra contained some irregularities. No evidence of OPS was found in *∆wbtI*. In keeping with these data, mass spectrometry analyses of sugar alditol acetates from whole bacteria digested with proteinase K, DNAase, and RNAase showed again that mannose, an LPS core sugar, was essentially absent from the *∆kdtA* (Figure S1). This demonstrated that this observation was not an artifact of the LPS isolation method employed. In addition, mass spectrometry analyses showed that each strain had a common lipid A structure, and SDS-PAGE showed that there was no *O*-antigen linked to it in either *∆kdtA* or *∆wbtI* (Figures S2 and S3).

**Figure 4:**
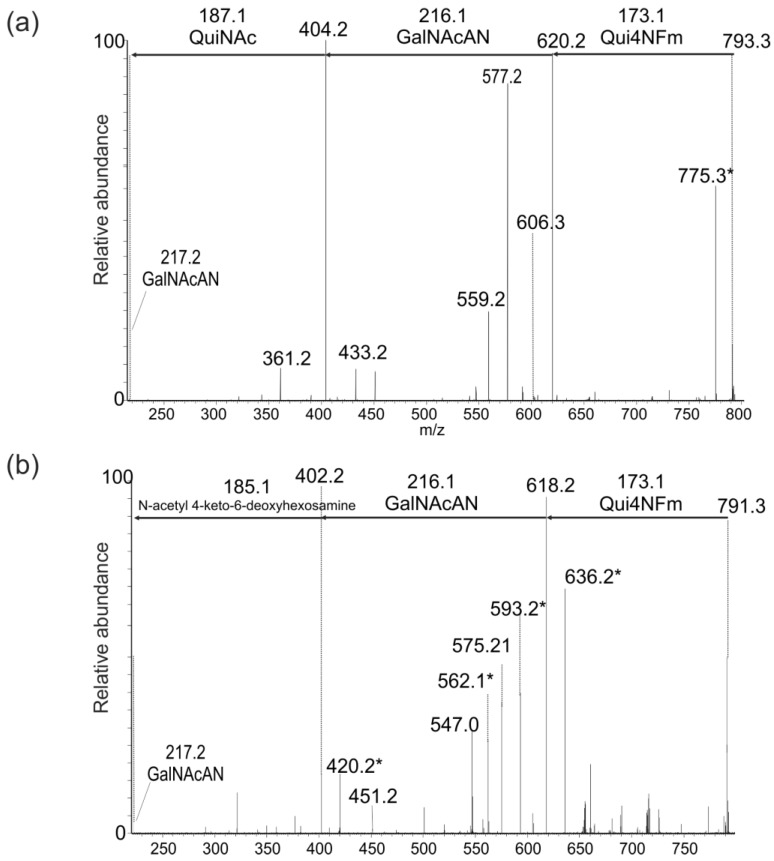
Positive ion ESI MSMS spectra of OPS. MS/MS spectra of *O*-polysaccharide (OPS) from (a) *Francisella tularensis* and (b) SCHU S4 *∆wbtC*.

Previous work by our group [6] has shown that the OPS repeat unit of *F. tularensis* has the composition QuiNAc (2-acetamido-2,6-dideoxy-D-glucose), Qui4NFm (4,6-dideoxy-4-formamido-D-glucose) and two moles of GalNAcAN (2-acetamido-2-deoxy-D-galacturonamide) or GalNAcAN- QuiNAc-GalNAcAN-Qui4NFm. An MS survey scan of OPS from SCHU S4 showed a prominent ion at m/z 793. MS/MS fragmentation of the monoisotopic ion at m/z 793 showed major fragment ions at m/z 620, 404 and 217 (Figure 4a). These corresponded to sequential losses of monosacchraides Qui4NFm (173 Da, m/z 793–620), GalNAcAN (216 Da, m/z 620–404), QuiNAc (187 Da, m/z 404–217), with the oxonium ion peak of GalNAcAN observed at m/z 217. Several minor fragment ions corresponded to disaccharide components (for example m/z 433, 216–216) or losses of water (m/z 775).

The mass spectrum of SCHU S4 *∆kdtA* OPS gave an identical fragmentation pattern to that of wild type OPS (data not shown). By contrast, the mass spectrum of the *∆wbtC* OPS had a peak at m/z 791 and a peak of much lower intensity at m/z 793. MS/MS of the ion at m/z 793 showed an identical fragmentation to that of wild type OPS. In contrast, the MS/MS spectrum of the ion at m/z 791 showed a different fragmentation pattern compared to that of the wild type OPS (Figure 4b). Of particular note, many of the fragment ions were shifted by -2 amu, compared to the wild type mass spectrum (notably m/z 402, 618) (Figure 4b). Sequential losses of Qui4Fm and GalNAcAN were observed, giving rise to fragment ions at m/z 618 and 402. Differing from wild type OPS, a loss of 185 Da was observed (m/z 402–217) instead of 187 (QuiNAc). Other OPS fragment ions in the *∆wbtC* mass spectrum were also 2 amu less than corresponding fragments in the wild type OPS spectrum (notably m/z 402, 618, 791). Interestingly, these major fragment ions in the *∆wbtC* spectrum were also accompanied by fragment ions +18 amu greater, likely corresponding to a hydrated form of the keto-group of *N*-acetyl 4-keto-6-deoxyhexosamine (m/z 420, 636, 809). Taken together, these data indicate that OPS of SCHU S4 *∆wbtC* is comprised of Qui4Fm-GalNAcAN-unknown sugar- GalNAcAN. The unknown sugar of mass 185 Da differs from QuiNAc by the loss of the mass of two hydrogen ions, therefore likely corresponds to the addition of *N*-acetyl 4-keto-6-deoxyhexosamine (185 Da) instead of *N*-acetyl 6-deoxyhexosamine (QuiNAc, 187 Da), *N*-acetyl 4-keto-6-deoxyhexosamine is the precursor of QuiNAc. 

**Figure 5:**
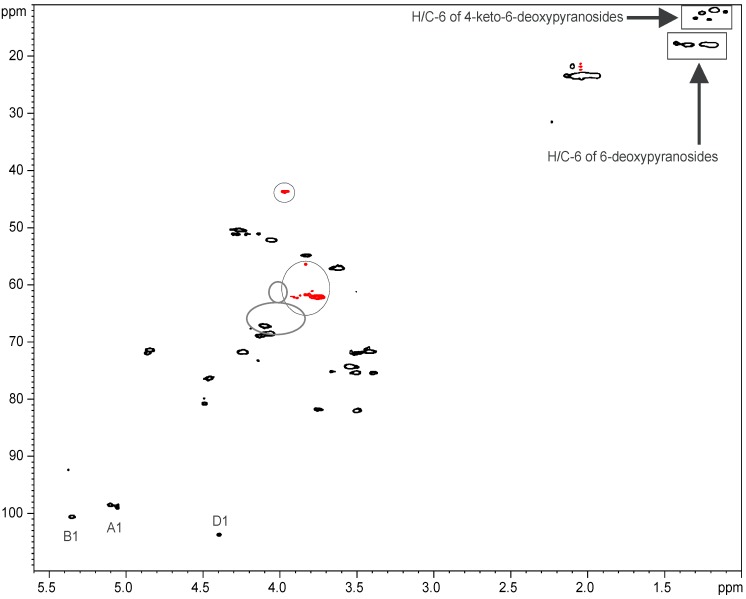
HSQC spectrum of *∆wbtC* OPS. Circled signals (CH_2_ groups) belong to impurities.

Next, *∆wbtC* OPS was analyzed using 2D NMR methods (Figure 5). The data supported the mass spectrometry analyses, confirming the presence of *N*-acetyl 4-keto-6-deoxyhexosamine in this mutant. The HSQC spectrum contained a signal at 1.17/11.8 ppm (H/C), characteristic for N-acetyl 4-keto-6-deoxyhexosamine. HMBC spectrum showed that C-4 of this monosaccharide gives a signal at 95.0 ppm, typical for a hydrated keto group. H/C-5 was found at 3.54/74.6 ppm. TOCSY spectrum showed only one correlation from methyl group signal at 1.17 ppm, which agreed with the absence of a proton at C-4. These data were in close agreement with those presented by others [17] describing *N*-acetyl 4-keto-6-deoxyhexosamine. 

### 2.2. Virulence and Immunogenicity of Mutants

**Figure 6:**
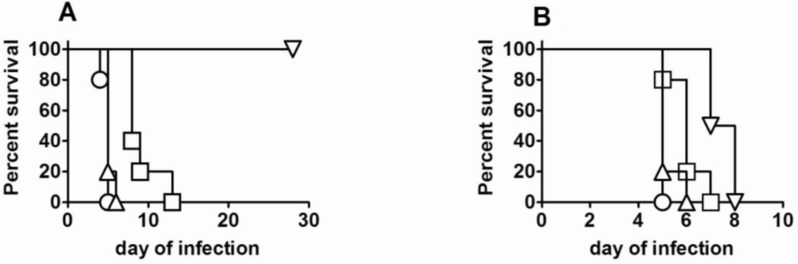
Survival of mice immunized with mutant strains to challenge with wild type SCHU S4. Naïve mice (circles), mice immunized with *∆kdtA* (squares), *∆wbtI* (triangle), *or ∆wbtC* (inverted triangle) were challenged six weeks later with (A), 1000 CFU SCHU S4 ID or (B), a low dose aerosol of ~ 20 CFU SCHU S4, and their survival was monitored.

The lethal dose of wild type SCHU S4 for mice by either the ID or aerosol route is <10 CFU, which kills them within 7 days [14,18]. In a previous preliminary study, we showed that the ID LD50 of *∆wbtI* or *∆kdtA* for BALB/c mice was >10^3^ CFU, and mice that recovered from this challenge were not protected against a subsequent ID or aerosol challenge with SCHU S4 [3]. We extend these finding in the present study wherein BALB/c mice were challenged ID with up to 10^7^ CFU of one or other mutant strain. All mice survived challenge with *∆wbtI* or *∆kdtA*, but 3/5 mice died after challenge with 10^7^ CFU of *∆wbtC*. Mice immunized with *∆wbtI* or *∆kdtA* displayed no overt inflammation at the puncture site, and remained healthy throughout. In contrast, mice immunized with 10^7^ CFU of *∆wbtC* developed large areas of necrosis at the site of inoculation, and the mice that died became severely sick beforehand. Mice immunized with 10^3^ or 10^5^ CFU of *∆wbtC* all survived. Some of the mice that survived ID inocula were subsequently challenged ID with 1000 CFU (>100 LD50) of wild type SCHU S4. All naïve mice and all the mice immunized with 10^7^ CFU of *∆wbtI* died by day 5 of challenge. The median time to death of mice immunized with 10^7^ CFU of *∆kdtA* was 8 days and this was significantly longer than the controls (P = 0.003 by Log-rank test). In contrast, 100% of mice immunized with 10^3^ of *∆wbtC* survived this challenge (Figure 6). Mice that survived immunization with 10^7^ CFU of one or other mutant were challenged with a low dose aerosol (~20 CFU) of SCHU S4. Naïve mice all died by day 5, all mice immunized with *∆wbtI* died between days 5–6, all mice immunized with *∆kdtA* died between days 5–7, and the 2 mice that survived ID immunization with 10^7 ^CFU of *∆wbtC* died on days 7 and 8. The latter two groups survived significantly longer than naïve mice (P = 0.014). 

### 2.3. Involvement of WbtC in Protein Glycosylation

Because *∆wbtC* appeared to have essentially normal LPS, its degree of attenuation was unexpected. Therefore, we examined the proteome of SCHU S4 *∆wbtC* by 2D-PAGE for evidence of other defects. 2D-PAGE has the advantage of readily showing protein isoforms differing by mass or isoelectric point and is amenable to staining with glycoreactive stains, making rapid differences in protein glycosylation readily discernible. 

**Figure 7:**
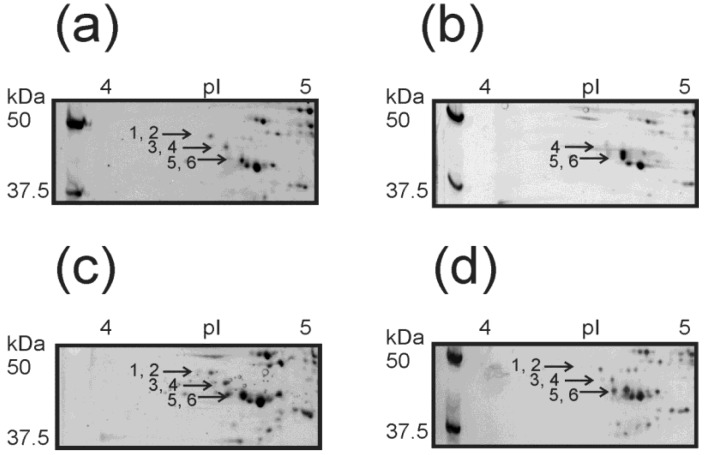
.2DE gels of *F. tularensis* strains *(a)*, *wild type SCHU S4*, *(b)*, *∆wbtC*,(c), *∆wbtI*, (d), *∆kdtA.* Arrowsmark isoforms 1-to-6 of glycoprotein DsbA.

We found differences in protein levels and glycosylation in previously reported glycoproteins, when comparing the proteome of *∆wbtC* and the other three strains. For example, we have previously characterized in detail the carbohydrate modification of a disulfide isomerase, DsbA [19] and shown that the protein migrates on 2D-PAGE to six distinct protein spots (Figure 7a) Glyco-staining showed that spots annotated 1–4 in Figure 7a, but not spots 5 and 6 were glycosylated (data not shown). We speculate that isoforms 1–4 likely correspond to varying degrees of protein glycosylation. The mutants *∆wbtI* and *∆kdtA* showed a similar distribution of isoforms of DsbA as SCHU S4 (Figure 7c and 7d). Interestingly, the *∆wbtC* mutant did not elaborate the same isoforms of DsbA as the other three strains; protein spots annotated as 1–3 were not observed in the *∆wbtC* mutant (Figure 7b). However, three spots (denoted 4–6 in Figure 7) were visible in the *∆wbtC* mutant, and migrated to almost identical MW/pI as those observed in SCHU S4. In particular, spot 6 migrated to a molecular mass of 38.5 kDa/pI 4.8, close to the values predicted from the translated gene sequence of DsbA. The identity of this protein was confirmed as DsbA, by tandem mass spectrometry of the in-gel tryptic digest and the absence of detectable glycopeptides strongly suggests that this corresponds to the unglycosylated form of the protein. Two protein spots of low abundance, were also observed in the *∆wbtC* mutant, migrating to MW/pI values of 41.5 kDa/pI 4.75 (spot 5) and 43.0 kDa/ pI 4.75 (spot 4). Tandem mass spectrometry of the tryptic digests of each spot from the *∆wbtC* mutant confirmed the spots to be DsbA, and MS/MS spectra showed very low levels of a peptide, modified via *O*-linkage with the previously observed 1156 Da glycan moiety [19]. Measurement of the comparative protein spot intensity between SCHU S4 and the *∆wbtC* mutant showed spots 5 and 6 to be present at similar levels in SCHU S4 and the *∆wbtC* mutant. The only glycosylated form of the protein observed in the *∆wbtC* mutant, spot 4, was present at approximately 3 fold lower levels compared with SCHU S4. Further analysis revealed only a few differences in the proteome of SCHU S4 *∆wbtC* by 2D-PAGE when compared to wild type. This included differential glycosylation of another glycoprotein reported to harbor the same modification, the pilin subunit PilA [20]. The protein migrates to several protein spots on 2D-PAGE, with molecular masses/isolectric points of 17.6 kDa/ pI 3.5, 17.6 kDa/ pI 3.9, 15.8 kDa/pI 3.6, 15.8 kDa/pI 3.95. Typically, we have observed all glycoforms to react with glycostain, however very weakly with protein staining reagents such as silver. In all but the *∆wbtC* mutant we observed the same profile of PilA glycoreactivity compared with wild type (data not shown). By comparison the *∆wbtC* mutant showed comparable area of observable reactivity with glycostain and no detectable protein stain. This strongly suggested that the glycosylation of PilA was negatively impacted by the deletion of the *wbtC* gene. Due to the weak reactivity of the protein isoforms of PilA, the comparative protein intensities were not measured. Other reported glycoproteins [21] were not detected in our hands using 2D-PAGE and glycostain.

## 3. Discussion

The ability to elaborate a long chain OPS is key to the virulence of *F. tularensis* subsp *tularensis* [8] and is confirmed herein. The OPS gene locus was annotated (*wbtA*-to-*wbtN*) and putative gene functions were assigned based upon homology to genes from other bacteria known to be involved in LPS biosynthesis [9]. In particular, many of the genes within the *F. tularensis* OPS locus were found to be homologous to the *O*-antigen genes of *Pseudomonas aeruginosa* serotype O6, which expresses a similar *O*-antigen repeat structure [22]. 

With particular relevance to the current study, the *F. tularensis* *wbtC* gene product has high homology to bacterial UDP-glucose 4–epimerase enzymes, especially *wbpV* of *Pseudomonas aeruginosa*. *WbpV* has been shown to have a role in UDP-QuiNac biosynthesis [23]. Similar to many epimerase/dehydratase enzymes, *wbtC* has putative NAD-binding domains which are thought to be important for dehydratase activities [24]. The *wbpV* null mutant in *P. aeruginosa* was deficient in B-band LPS production [23]. This does not appear to be the case in SCHU S4, where deletion of the *wbtC* gene still results in an *O*-antigen repeat structure visualised by 1D-PAGE, albeit with a somewhat different banding pattern to that observed in wild type LPS. A shift in LPS electrophoretic mobility was previously observed in a *wbtC* mutant of *F. tularensis* LVS [13]. A more recent study showed that a monoclonal antibody specific for wild type OPS failed to recognize the *O*-antigen expressed by the LVS *wbtC* mutant [25] implying that the OPS from the latter had altered structure. 

The sugar detected as a component of the SCHU S4 *∆wbtC* OPS in the current study, N-acetyl 4-keto-6-deoxyhexosamine, is a biosynthetic intermediate of the QuiNAc pathway, and its presence suggests a mutation in the enzyme reducing UDP-4-keto-product into UDP-QuiNAc. By MS, two peaks were detected in *wbtC* OPS, the minor peak (m/z 793) corresponds to the presence of QuiNAc with the dominance of its precursor (major peak m/z 791), *N*-acetyl 4-keto-6-deoxyhexosamine. The former could result from the presence of another 4-reductase enzyme, involved in the biosynthesis of another deoxy sugar. Studies in other bacteria, for example *Rhizobium etli* showed that mutation of the sugar epimerase/reductase resulted in replacement of QuiNAc in LPS with its 4-keto derivative [26]. The latter study also detected low levels of QuiNAc in the OPS. 

Several recent reports have described the modification of several *Francisella* proteins with glycan moieties in different subsp [19,20,21,27], with reports of putative roles of genes within the O-antigen biosynthetic locus [27] and other putative biosynthetic gene clusters [19]. Interestingly, *∆wbtC* also showed an absence or marked decrease in abundance of the glyosylated isoforms of the DsbA homologue, which others have shown to be a putative lipoprotein [28]. The same group showed that deletion of the *dsbA* gene from SCHU S4 resulted in complete attenuation. Several recent publications show that glycoproteins of type A and B strains are modified with a 1156 Da glycan moieity [25,26,27]. These prior works have shown that a PglA homologue [25] and LPS biosynthetic genes *wbtDEF* [27] are putatively involved in protein glycosylation in *Francisella*. Here we show a role for *wbtC* in protein glycosylation. The utilization of common enzymes in LPS biosynthesis and protein glycosylation is not unique to *F. tularensis*. For instance, others have shown that *P. aeruginosa* [29] and *Aeromonas caviae* [30] use the same transferase in LPS biosynthesis and protein glycosylation to incorporate a common sugar. Additionally, *Helicobacter pyloris was* recently shown to use a homolog of a protein glycosylation transferase in its OPS biosynthesis [31]. It was also recently shown [32] that *Campylobacter jejuni* uses the same enzyme to modify its LPS and flagella rod protein. However, MS indicates that OPS and *F. tularensis* glycan do not contain any common sugars by monosaccharide mass (not shown). Thus, the basis for the involvement of *wbtC* in both processes remains unknown; one possibility is that sugar precursors are drawn from the OPS biosynthetic pathway into the glycosylation pathway for DsbA. In this regard, it is interesting to note, that *F. novicida* does not possess the *wbtC* gene [8], and its DsbA protein homologue appears to be glycosylated with different sugars than those found in the *F. tularensis* protein [19]. Interestingly, glycosylation of DsbA in the *wbtC* mutant was not completely abolished, with observable low levels of modification with the same glycan chain. This suggests that a second enzyme with homology to WbtC is participating in protein glycosylation. Previous work identified a second glycan biosynthetic gene cluster, encompassing the FTT0789-FTT0800 [26]. One of the genes, within the second polysaccharide gene cluster, FTT0791, is a putative UDP-Hex epimerase with some homology to WbtC. When the corresponding gene was disrupted, the mutant strain showed no detectable presence of modified form of the protein [26]. Some bacteria have been reported to harbor several copies of these epimerase genes; it is likely that this enzyme may also have some activity in protein glycosylation. Indeed, there is much that remains to be deciphered regarding the mechanisms of protein glycosylation in Francisella, with reports of PglA homologue responsible for glycosylation in several strains [20,27]. 

The *wbtI* gene product has high homology to sugar transamine/perosamine synthetases and was proposed to be involved in the biosynthesis of the fourth sugar in the *F. tularensis* *O*-antigen repeat, Qui4Fm [9], specifically, in amination of the sugar moiety. In this study, deletion of the gene resulted in lack of detectable *O*-antigen reactivity by 1D-PAGE. Another study generated a mutant of LVS with a single amino acid substitution in the wbtI gene (WbtIG191V). This resulted in disruption of *wbtI* enzymatic activity, and loss of OPS, including inability to detect the *O*-antigen specific sugar QuiNAc [11]. The *kdtA* gene has high homology to 3-deoxy-D-manno-octulosonic-acid transferase enzymes and harbours two conserved glycosyltransferase domains. In *E. coli*, KdtA is the enzyme responsible for the attachment of the two 3-deoxy-D-manno-octulosonic acid residues to lipid A. The core sugars and OPS are then added to KDO2-lipid A, to yield mature LPS [33]. Our analyses showed that OPS is still synthesized by the SCHU S4 *∆kdtA* mutant, but mature LPS is not formed. Recently, others have shown that *F. tularensis* possesses a microcapsule consisting of polymerized O-antigen [25]. However, the serum agglutination results reported herein, suggest that the *O*-antigen found in the *∆kdtA* mutant is not expressed at the surface. 

Others have previously shown that Francisella mutants that fail to elaborate *O*-antigen are much more susceptible than wild type bacteria to being killed by normal human serum [34,35,36], in particular the, OPS must be conjugated to Lipid A-core in order to protect the pathogen from serum-mediated killing. Herein we observed that mice that recovered from infection with mutant *∆wbtC* were fully protected against a subsequent intradermal challenge with wild type SCHU S4, whereas mice infected with *∆kdtA* were partially protected and those infected with *∆wbtI* were completely unprotected from such challenge. Previously, our group has shown that dual vaccination with an *O*-antigen deletion mutant of LVS and an *O*-antigen conjugated to tetanus toxoid, but not with either material alone could protect mice from systemic challenge with SCHU S4 [18]. We hypothesize that the *kdtA* mutant has partially replicated this effect because it produces unconjugated *O*-antigen, unlike *wbtI* which fails to elaborate it at all. None of the SCHU S4 mutants elicited any significant protection against aerosol challenge when administered via the skin, the sole route of administration recommended for vaccinating humans with LVS.

## 4. Experimental Section

### 4.1. Bacterial Strains

Wild type SCHU S4 was originally isolated from a human patient [37]. For the present study it was obtained from a stock (FSC237) maintained at Umea University, Sweden. Mutants *ΔwbtI* and *ΔkdtA* have been described previously [3]. An in-frame deletion of *wbtC* was generated by allelic exchange using the previously described method [3]. For the present study, stock cultures of all strains were prepared by growing them as confluent lawns on cystine heart agar supplemented with 1% (w/v) hemoglobin (CHAH). Bacteria were harvested after 48 h incubation at 37 °C into freezing medium consisting of modified Mueller Hinton broth containing 10% w/v sucrose [38]. Stocks were aliquotted in volumes of 1 mL and stored at −80 °C at a concentration of 10^9^–10^11^ CFU/mL. 

### 4.2. Isolation and Analysis of LPS

LPS and free polysaccharides were isolated from plate-grown bacteria by standard methods that we have previously used for *F. tularensis* and *F. novicida* [5,16]. Briefly, cells were extracted with 45% phenol for 30 min at 80 °C, dialyzed, nucleic acids precipitated by addition of AcOH up to 10% and centrifugation at 5000 g. Solutions were exhaustively dialyzed against water and freeze dried. For gas chromatography, the resulting LPS or polysaccharde was hydrolyzed with 3M trifluroacetic acid (120 °C, 3h) and sugars converted to alditol acetates by standard methods. For NMR analysis, LPS or polysaccharides were hydrolyzed with AcOH and *O*-chain isolated by gel chromatography as previously described [5]. ^1^H and ^13^C NMR spectra were recorded using a Varian Inova 500 MHz spectrometer for samples in D_2_O solutions at 25–45 °C with acetone internal reference (2.23 ppm for ^1^H and 31.5 ppm for ^13^C) using standard pulse sequences double quantum filtered correlation spectroscopy (DQCOSY), total correlation spectroscopy (TOCSY) (mixing time 120 ms), nuclear overhauser effect spectroscopy (NOESY)(mixing time 400 ms), heternuclear single quantum coherence (HSQC) and heternuclear multiple bond correlation (HMBC) (80 ms long range transfer delay). 

Mass spectrometry analyses were carried out using an LTQ Orbitrap XL mass spectrometer (Thermo, Waltham, MA, USA). *O*-antigen solutions were diluted two-fold with a solution of 25% methanol, 1% formic acid and infused into the mass spectrometer at 1 µL/min. MS/MS spectra were recorded on putative glycan related fragment ions under the following conditions: RF lens 1 120 V, CE 28. Resolution was typically 80,000 (50% valley definition). Collision-induced dissociation MS/MS analysis was performed on the glycan ions to confirm their identity.

### 4.3. De-O-Acetylation of Cell Samples

Bacterial strains were harvested from growth on CHAH and killed with 2% phenol for a minimum of two hours. The cell pellets were then harvested by centrifugation for 20 mins at 13,0000 *g* at 7 °C. Cell pellets were water washed and spun again before removing supernatant and lyophilizing. Lyophilised samples were treated with 0.35 mg/mL proteinase K for 2 hours at 37 °C and 65 °C for 10 minutes. Samples were lyophilized again and treated with 10 uL of 0.1 mg/mL RNAase and 0.2 mg/mL of DNAase in 20 mM ammonium acetate. After 4 hours at 37 °C, samples were lyophilized once more and a protion removed for 1D-PAGE and mass spectrometry studies. To the remainder of the sample, 200 uL of hydrazine was added under nitrogen. Samples were incubated at 37 °C for 1.5 hours before quenching on dry ice with acetone. Samples were pelleted using a benchtop centrifuge, supernatant discarded and the pellet resuspended in acetone. Mass spectrometry studies were carried out using an ABSciex 4700 MALDI TOF/TOF mass spectrometer. 

### 4.4. 1D-PAGE and Western Blotting Analysis of LPS

Lyophilised LPS was solubilised in Milli-Q and mixed with Laemmlli buffer prior to separation by SDS-PAGE (12% acrylamide). Gels were transferred to PVDF membranes, as described in our recent work [39] and LPS was visualised by immunoblotting with a 1:2000 dilution of polyclonal antiserum obtained from mice immunised with BSA-conjugated *O*-antigen [6]. The secondary antibody, HRP conjugated goat anti-mouse IgG, (Perkin-Elmer Life and Analytical Sciences, Woodbridge, Ontario, Canada) was used at a dilution of 1:5000. Reactivity was visualised using the ECL kit (GE Healthcare) as per the manufacturer’s instructions. Material extracted from whole killed cells was separated using 1D-PAGE as described and visualized using the Pro-Emerald Q LPS Glycostain kit (Invitrogen), as per the manufacturer’s instructions. Stained gel images were captured using a FluorS Scanner (Biorad, Hercules, CA, USA) and PDQuest software (Biorad, Hercules, CA, USA).

### 4.5. 2D-PAGE Analysis

Francisella strains were plated for single colony growth on CHAH. At 72 h of incubation, 200 colonies of one or other strain were resuspended in 12 times the estimated pellet volume of lysis solution (7 M urea, 2 M thiourea, 1% (w/v) DTT, 4% (w/v) CHAPS and 0.5% (w/v) ASB-14 [40]). Protein concentrations of the extracts were assay using the RC-DC protein assay (Biorad, Hecules, CA, USA) or using a modified Bradford Assay (Biorad, Hecules, CA, USA). Whole cell lysates were separated using immobilised pH gradient strips (IPG), either linear pH 4–7, 17 cm (Biorad, Hercules, CA, USA). 100 µg of each protein solution was diluted with the lysis solution, with 0.5% v/v pH 3–10 Biolytes (Biorad, Hercules, CA, USA) and 0.003% Orange G (Biorad, Hercules, CA, USA). Proteins were loaded onto the IPG strips by in-gel rehydration overnight. Isoelectric focusing was conducted as described previously [40]. The second dimension was carried out with 12% polyacrylamide gels (190 × 190 × 1.5 mm) using the Protein IIxi System (Biorad, Hercules, CA, USA) at 24 mA per gel for 5 hours. Gels were first stained with Emerald Q Glycostain (Invitrogen, Burlington, ON, Canada) to visualise glycoproteins and subsequently with Sypro Ruby for protein visualisation. Stained gel images were captured using a FluorS Scanner (Biorad, Hercules, CA, USA) and PDQuest software (Biorad, Hercules, CA, USA).

### 4.6. Protein Identification

Spots on 2DE gels were excised, digested with trypsin, and the resulting peptides analysed by nano-electrospray tandem mass spectrometry (nLC-MS/MS) as recently described [41]. The peaklist files of MS2 spectra of the excised protein spots were searched against the NCBI *F. tularensis* database (2007.09.05) with 12283 entries using the MASCOT^TM ^search engine (version 2.2.0) (Matrix Science, London, UK) for protein identification. The mass tolerance for precursor ions was ± 0.8 Da and the mass tolerance for fragment ions was ±0.15 Da with trypsin. A cut-off ions score of 30 was used to indicate identity and in addition, all spectral matches were verified manually. Unmatched MS2 spectra were examined manually to determine the sequence of peptide y and b type ions.

### 4.7. Biological Studies

Female BALB/c mice were purchased from Charles River Laboratories (St. Constant, Quebec) and entered experiments at 6–8 weeks of age. Mice were maintained and used in accordance with the recommendations of the Canadian Council on Animal Care Guide to the Care and Use of Experimental Animals. Intradermal (ID) inocula were injected into a fold of skin in the mid-belly in a volume of 0.05 mL saline. Aerosol challenges were performed using an InTox Products nose-only exposure chamber as previously described [42]. Survival curves were constructed using GraphPad Prism 5 software (Graphpad Software, La Jolla, CA) and compared for statistical significance by Log-rank test using GraphPad Prism 5 software. To test the susceptibility of the mutants to serum, they were suspended in Chamberlains Defined Medium [43] in the presence or absence of 10% unheated normal human serum. Colony counts were performed a 0 and 4h. For the bacterial agglutination assay, 20 μL of culture suspended in saline was admixed with 5 μL of Francisella-specific rabbit antiserum (Difco, BD Biosciences, Mississauga, ON, Canada).

## 5. Conclusions

In the present study, we show that deletion of select genes from the *Francisella tularensis* subsp *tularensis* LPS biosynthetic locus results in not only changes in LPS structure, but more subtle effects upon protein glycosylation and bacterial virulence. In particular, deletion of the *wbtI* gene from SCHU S4, results in an avirulent mutant that does not express OPS, and fails to elicit a protective immune response. In contrast, deleting gene *wbtC*, led to a more subtle effect on OPS and also interfered with glycosylation of several known glycoproteins. This mutant retained slight virulence and elicited full protection against systemic, but not aerosol challenge with wild type SCHU S4. We show too that a mutant missing a homolog of a gene, *kdtA*, that encodes KDO transferase, lacked an obvious core region and produced free polymeric OPS that did not appear to be elaborated at the surface of the pathogen. It was avirulent, but elicited partial protective immunity against systemic challenge with wild type bacteria. This work contributes to a broader understanding of the complexity of the biology of this bacterium, and underlines the work still required for the design of an effective live vaccine. 

## Supplementary Files

Supplementary File 1 Supplementary Materials (PDF, 472 KB)
