# Supplementary material for: Roles for wbtC, wbtI, and kdtA Genes in Lipopolysaccharide Biosynthesis, Protein Glycosylation, Virulence, and Immunogenicity in Francisella tularensis Strain SCHU S4
Source: Pathogens. 2012 Sep 10;1(1):12–29. doi: 10.3390/pathogens1010012 (PMC4141488; doi:10.3390/pathogens1010012)

Supplementary Materials

Figure S1. GC-MS analysis of alditol acetates of (**a**) SCHU S4, (**b**) Δ*wbtC*, (**c**) Δ*kdtA* and (**d**) Δ*wbtI*, obtained after hydrolysis of material remaining after treatment of whole killed bacteria with DNase, RNase and proteinase K. The Δ*wbtC* spectrum was similar to wild type *F. tularensis*. Of note the Δ*kdtA* mutant had low levels of the LPS core sugar mannose, evidence that this mutant does not elaborate a core structure. An arrow on panel (c) shows the absence of mannose in the Δ*kdtA* mutant. The Δ*wbtI* mutant had levels of the mannose comparable to those observed in the wild type strain, however did not have detectable levels of other sugars, such as QuiN. These data are similar to those obtained from GC-MS analyses of material isolated by LPS preparations.

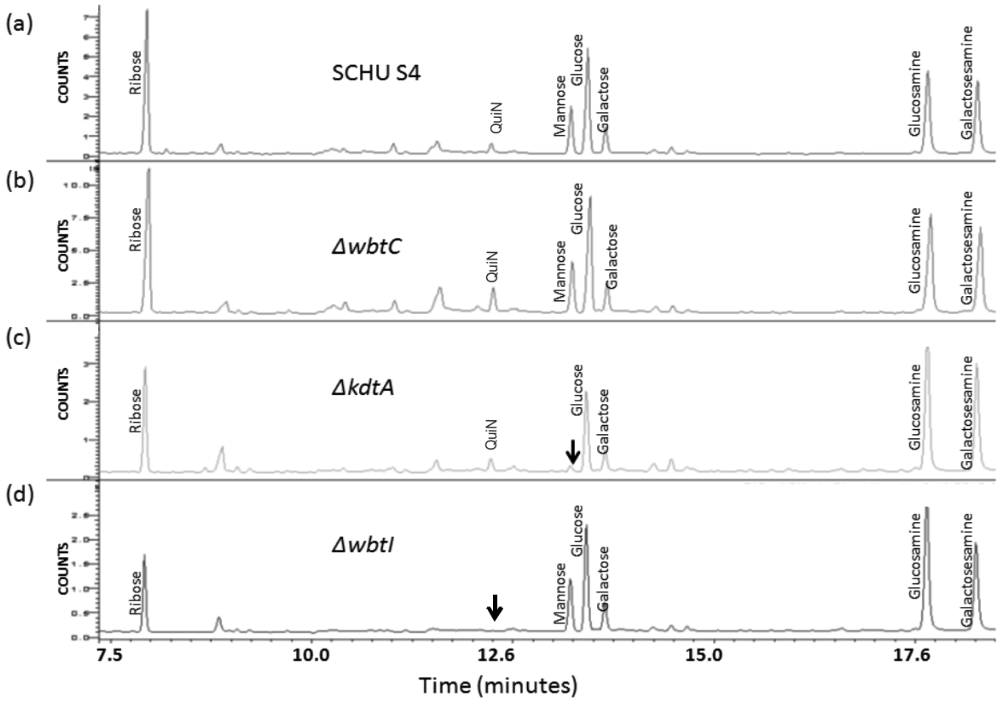

Figure S2. MALDI-TOF analysis of purified lipid A from (**a**) SCHU S4, (**b**) Δ*kdtA*, (**c**) Δ*wbtC* and (**d**) Δ*wbtI*. As reported by others (Schilling, McLendon et al. 2007), species were observed at m/z 1665.3 and 1504.2, with a putative composition of $C_{16:0}$, 3 × $C_{18:0}$(3-OH), phosphate and $GalN_{n=1/0}$. No differences in the fragment ion profiles of lipid A isolated from wild type and each mutant was observed.

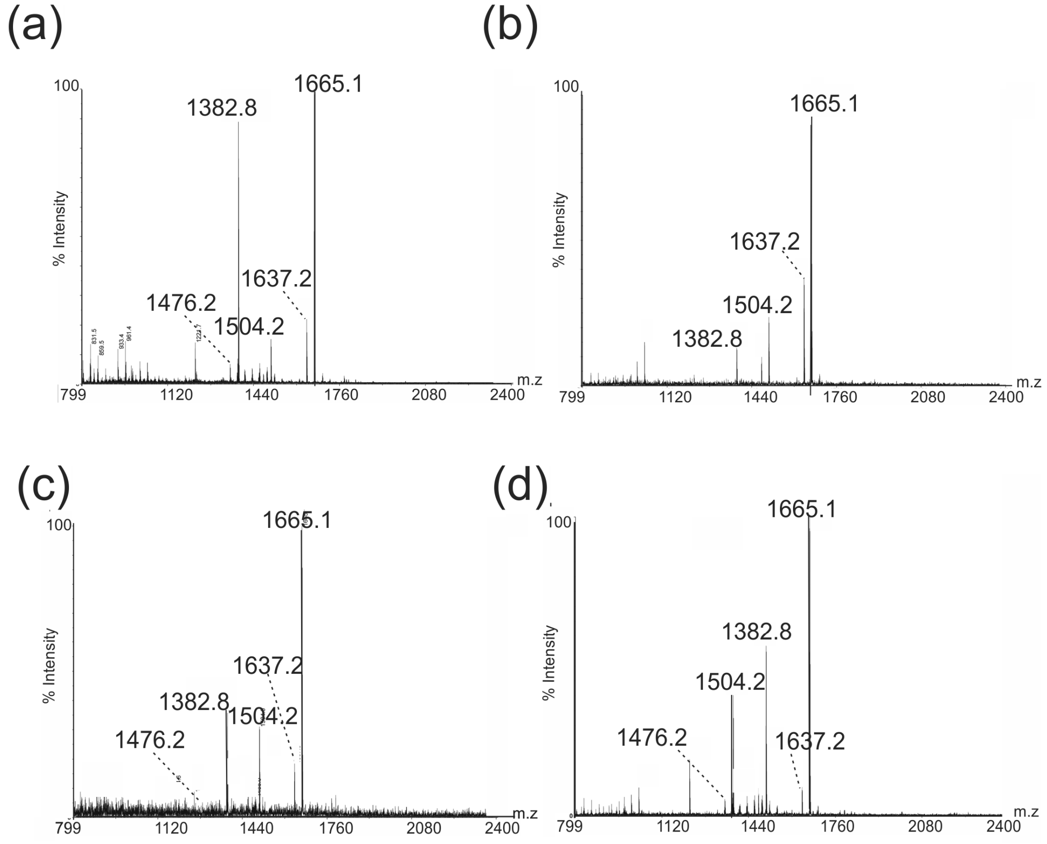

Figure S3. 1D-PAGE of killed whole bacterial cells treated with DNase, RNase and proteinase K, visualised with periodic acid based glycostain. Candy Cane marker (Lane 1), SCHU S4 (lane 1), Δ*wbtC* (lane 2), Δ*wbtI* (lane 3) Δ*kdtA* (lane 4). The LPS and unconjugated lipid or carbohydrate portions of cells were visualised using a commercial glycostaining kit, based upon traditional periodic acid chemistry, and a fluorescent marker group. O-antigen banding pattern was observed in cell extracts of wild type and Δ*wbtC* only.

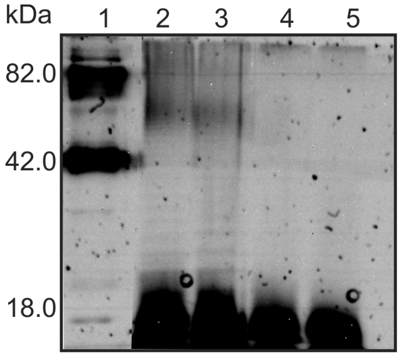

Supplement: Supplementary File 1 — Supplementary Materials (PDF, 472 KB) [file pathogens-01-00012-s001.pdf]
